# Nurses’ perceptions of barriers and supportive behaviors in end-of-life care in the intensive care unit: a cross-sectional study

**DOI:** 10.1186/s12904-022-01020-4

**Published:** 2022-07-19

**Authors:** Dan-dan Xu, Dan Luo, Jie Chen, Ji-li Zeng, Xiao-lin Cheng, Jin Li, Juan-juan Pei, Fen Hu

**Affiliations:** 1 Department of Critical Care Medicine, Zhongnan Hospital of Wuhan University, Clinical Research Center of Hubei Critical Care Medicine, Critical Care and Anesthesia Nursing Research Center, School of Nursing, Wuhan University, PO Box 430071, No. 169 Donghu Road, Wuhan, Hubei Province China; 2 Wuhan University School of Health Sciences, Wuhan, China; 3 University of Connecticut School of Nursing, Mansfield, USA; 4 Department of Critical Care Medicine, Zhongnan Hospital of Wuhan University, Clinical Research Center of Hubei Critical Care Medicine, Wuhan, China; 5 Zhongnan Hospital of Wuhan University, Wuhan, China

**Keywords:** End-of-life care, Intensive care unit, Nurses, Barriers, Supportive behaviors

## Abstract

**Background and aim:**

Patient deaths are common in the intensive care unit, and a nurse’s perception of barriers to and supportive behaviors in end-of-life care varies widely depending upon their cultural background. The aim of this study was to describe the perceptions of intensive care nurses regarding barriers to and supportive behaviors in providing end-of-life care in a Chinese cultural context.

**Methods:**

A cross-sectional survey was conducted among intensive care nurses in 20 intensive care units in 11 general hospitals in central and eastern China. Instruments used in this study were general survey and Beckstrand’s questionnaire. Data were collected via online survey platform. Descriptive analysis was used to describe general characteristics of participants and mean and standard deviations of the barriers and supportive behaviors. The mean and standard deviation were used to describe the intensity and frequency of each barrier or supportive behavior following Beckstrand’s method to calculate the score of barriers and supportive behaviors. Content analysis was used to analyze the responses to open-ended questions.

**Results:**

The response rate was 53% (*n* = 368/700). Five of the top six barriers related to families and the other was the nurse’s lack of time. Supportive behaviors included three related to families and three related to healthcare providers. Nurses in the intensive care unit felt that families should be present at the bedside of a dying patient, there is a need to provide a quiet, independent environment and psychological support should be provided to the patient and family. Nurses believe that if possible, families can be given flexibility to visit dying patients, such as increasing the number of visits, rather than limiting visiting hours altogether. Families need to be given enough time to perform the final rites on the dying patient. Moreover, it is remarkable that nurses’ supportive behaviors almost all concern care after death.

**Conclusions:**

According to ICU-nurses family-related factors, such as accompany of the dying patients and acceptence of patient’s imminent death, were found the major factors affecting the quality of end-of-life care. These findings identify the most prominent current barriers and supportive behaviors, which may provide a basis for addressing these issues in the future to improve the quality of end-of-life care.

**Supplementary Information:**

The online version contains supplementary material available at 10.1186/s12904-022-01020-4.

## Introduction

End-of-life care involves a multidisciplinary team (physicians, nurses, family members, social workers, pastors, etc.) that provides physical, psychological, social and spiritual assistance to dying patients and their families [1]. end-of-life care aims to help patients die in comfort, peace and dignity and has been implemented widely in oncology settings. The intensive care unit (ICU) is another setting with a high patient mortality rate. A study in 84 countries showed that ICU mortality rates ranged from 9.3 to 26.2% [2]. In mainland China, this rate was 26.0 to 45.6% from 2005 to 2016 [3]. In addition, patients suffering pain due to their physical status and various invasive treatments, such as endotracheal intubation and tracheotomy [4]. Many dying patients often lack privacy and their wishes are not respected, so their dignity is lost [5, 6]. Moreover, they lack family companionship in the ICU [7].

End-of-life care in ICU is important. End-of-life care is helpful to improve the quality of life of the terminal patients, relieve their pain, and alleviate the emotional reactions (such as sadness, depression, angry, fear, and shame.) of the family members when facing and dealing with death [8]. Studies have shown that end-of-life care can improve the quality of death, shorten the length of stay, and reduce the cost of hospitalization in ICU [9, 10].

Healthcare providers have begun to pay attention to patients’ desire for a good death and have advocated for the implementation of end-of-life care in the ICU [11]. However, there are many factors that affect implementation of end-of-life care, such as medical uncertainty, cultural differences and disagreements among healthcare providers [12, 13]. Behavioral factors that influence end-of-life care include positive supportive factors and negative impeditive factors [14]. Barriers create gaps between value and practice while supportive behaviors promote value and enhance practice. Timely identification of influencing factors is the basis to facilitate end-of-life care in an ICU. However, in China, healthcare providers have limited understanding of the barriers to and supportive behaviors of end-of-life care, especially in the ICU environment. As a member of a multidisciplinary team, ICU nurses spend more time with patients than other members. They act as an implementer, educator and a coordinator in end-of-life care [15].

Although there have been some studies of the barriers and supportive behaviors of end-of-life care globally [16–18], nurses from varied cultural backgrounds have different perspectives. In mainland China, public values are deeply influenced by Confucian culture. In a family-oriented system, the family is more important than the patient’s autonomy [19]. Death is not a personal issue but a family issue. Due to the concept of “filial duty”, family members may be inclined to prolong a patient’s life to avoid public criticism. Moreover, public discussion of death is considered inappropriate. Even in the same culture background, the health care system may make difference. A study conducted in Hong Kong reported the nurses’ perceptions of barriers andsupportive behaviors to end-of-life care [20], but the healthcare practices used there appear to align closer to those used in Europe compared with those used in mainland China. There are differences in the healthcare system and the medical treatment model in mainland China compared with Hong Kong. Additional barriers to implementation of end-of-life care in ICUs center on legal and procedural issues. In developed countries such as the United States, the United Kingdom and Japan, end-of-life care has been integrated into medical education, health insurance programs and relevant laws and policies to support end-of-life care [21–23]. In China, there is a lake of concensus on national guidelines on end-of-life care and the absence of a sound insurance system to support their implementation [24]. The Chinese legal system has no laws on end-of-life care and healthcare providers may feel exposed to legal risk when performing do-not-resuscitate orders [25].

To improve/facilitate end-of-life care in ICU, it’s critical to investigate ICU nurses’ perceptions of barriers and supportive behaviors of end-of-life care since nurses are the vital providers of end-of-life care in ICU. The aims of the study were: (1) to describe ICU nurses’ perceptions of barriers and supportive behaviors regarding end-of-life care; (2) to explore specific behaviors that are frequently reported as barriers and facilitators among these nurses.

## Methodology

### Design and setting

This study used a cross-sectional survey design in 20 ICU settings of 11 general hospitals in central and eastern China. These hospitals are located in large, economically developed cities and are part of the national healthcare system. They are considered tertiary-level hospitals which are explicity and heirachically defined, serving a broad geographic and population base. The ICU settings have established visiting hours, usually in the afternoon. Family members of a dying patient are normally asked to follow the visiting policy although some ICUs may deviate from this policy but family members are not allowed to remain at the bedside for extended periods.

### Sample

Nurse participants were recruited by convenience sampling. Inclusion criteria were:(1) registered nurse, (2) having a minimum of one year of work experience in an ICU, and (3) prior experience in caring for dying patients. Exclusion criterion was a nurse not providing direct care for patients.

### Instruments

The questionnaire consisted of two parts. The part one was a researcher-designed socio-demographic questionnaire, which include age, gender, education level, years of work experience, current position and number of dying patients cared for. The part two was a questionnaire on ICU nurses’ perceived barriers and supportive behaviors to end-of-life care.

The *National Survey of Critical Care Nurses’ Perceptions of End-of-life-Care* developed by Beckstrand [26] was used to identify barriers to and supportive behaviors for end-of-life care. It consists of 29 items focusing on barriers, 24 items on supportive behaviors and three open-ended questions. These questions include: (1) Describe any missing obstacles in detail. Indicate how large each obstacle is and how frequently it occurs; (2) Describe any missing supportive behaviors in detail. Indicate how large the support is and how frequently it occurs; (3) If you had the ability to change just one aspect of the end-of-life care given to dying ICU patients, what would it be? The questionnaire uses a six-point Likert-type scale to measure the intensity and frequency of barriers (0 = not an obstacle, 1 = extremely small, 2 = small obstacle, 3 = medium obstacle, 4 = large obstacle, 5 = extremely large); frequency scores (0 = never occurs, 1 = almost never occurs, 2 = sometimes occurs, 3 = fairly often occurs, 4 = very often occurs, 5 = always occurs). The supportive behaviors part is scored in the same way as the barrier part. Using the mean item score to determine the intensity and frequency of each item, the perceived intensity of each item is the mean of intensity multiplied by the mean of frequency. The higher the item’s perceived intensity score, the more prominent the barrier or supportive behavior becomes. Each item’s intensity mean is multiplied by the item’s frequency mean to obtain a perceived intensity score for the barrier or a perceived supportive behavior score for the supportive behavior.

The author of the original questionnaire was contacted to obtain permission to translate the survey into Mandarin. The questionnaire was forward-translated and back-translated from English into Mandarin by five bilingual researchers using Brislin’s protocol [27]. Six expert nurses with clinical experience in end-of-life care were invited to conduct a content validity test. Based on the test results, 3 barrier items and one supportive item were deleted from the original questionnaire as follows: (1) Physicians who are overly optimistic to the family about the patient surviving: Chinese physicians rarely appear overly optimistic for fear that the reality of the situation will not match expectations and lead to conflicts about medical care. (2) Family and friends who continually call the nurse wanting an update on the patient’s condition rather than calling the designated family member for information: In mainland China, it is common to provide family with the phone number of the ICU physician, not the ICU nurse’s station. (3) Unit visiting hours that are too liberal: ICU visiting hours are limited and strict and family are not allowed to visit at will. Letting the social worker or religious leader provide primary care of the grieving family: There are few social workers and religious leaders engaged in end-of-life care in China. The adjusted questionnaire has been approved by the original author. In this study, the content validity index was 0.915 for barriers and 0.963 for supportive behaviors. Regarding the reliability analysis of barriers and supportive behaviors, Cronbach’s alpha was 0.937 and 0.945, respectively; test-retest reliability was 0.083 and 0.816; and split-half reliability was 0.899 and 0.860.

### Data collection

Data were collected from January to March 2020. The purpose of this study had been previously explained to nurses in the selected hospitals. The survey was conducted on Wen Juanxing, a professional online questionnaire survey platform, which provides users with questionnaire design, data collection, etc. A web link was provided for nurses who agreed to participate and informed consent was obtained prior to completion of the survey questionnaire. Participation was voluntary and anonymity was maintained. Invitations to participate were extended to 700 nurses, and 368 participated. A total of 316 survey questionnaires were determined to be valid, for a response rate of 45%. Participants were not allowed to complete more than one survey questionnaire.

### Data analysis

Data were collated and analyzed using IBM SPSS 21.0. Descriptive statistics were used to analyze participants’ socio-demographic and questionnaire data, including mean value, standard deviation and percentage. The mean and standard deviation were used to describe the intensity and frequency of each barrier or supportive behavior following Beckstrand’s method to calculate the score of barriers and supportive behaviors. The perceived intensity score (PIS) mean for intensity multiplied by mean for frequency, and the perceived supportive behavior score (PSBS) mean for intensity multiplied by mean for frequency.

### Ethical and research approval

Ethical approval was obtained from Zhongnan Hospital of Wuhan University (Ethical Review Number: 2019104). Informed consent was obtained from participants and identifying information remained confidential and was available to only the researchers.

## Results

### Participants’ socio-demographic characteristics

Table 1 summarizes the general characteristics of the participants. Nearly 80.7% of the participants had a bachelor’s degree. The majority of participants (78.2%) were considered to be a junior nurse (According to the professional and technical levels, divided into junior, mid-level, senior). Half had more than five years of work experience. Approximately 73% of the participants had cared for more than ten dying patients sinece they started working.

**Table 1:** General characteristics of participants (*N* = 316)

Variables	n (%)
Age (in years)	
<20	1 (0.3)
20 ~ 30	190 (60.1)
30 ~ 40	113 (36.8)
>40	12 (3.8)
Education	
Specialist qualification	57 (18.0)
Bachelor’s degree	255 (80.7)
Master’s degree	4 (1.3)
Position	
Junior	247 (78.2)
Mid-level	63 (19.9)
Senior	6 (1.9)
Work experience in ICU (in years)	
<5	171 (54.1)
5 ~ 10	90 (28.5)
10 ~ 15	41 (13.0)
>15	14 (4.4)
Number of dying patients cared for	
<10	87 (27.5)
10 ~ 20	81 (25.6)
20 ~ 30	42 (13.3)
>30	106 (33.5)

### ICU nurses’ perceptions of barriers

The top six barriers were identified according to the results of PIS. Five barriers related to family members: “the nurse having to deal with distraught family members while still providing care for the patient (item 2)”; “the nurse having to deal with angry family members (item 21)”; “the family, for whatever reason, is not with the patient when he or she is dying (item 22)”; “families not accepting what the physician is telling them about the patient’s poor prognosis (item 1)”; and “family members not understanding what ‘life-saving measures’ really mean, i.e.. (item 19)” The sixth barrier was related to lack of time: “not enough time to provide quality end-of-life care because the nurse is consumed with activities trying to save the patient’s life (item 5)” The five items with the lowest perceived intensity score related to symptom management of dying patients and team members. The intensity and frequency rankings of the top six barriers were basically the same (see Table 2).

**Table 2:** ICU nurses’ perceived barriers to end-of-life care

Barrier	Frequency score			Intensity score			PIS
	Mean	SD	Rank	Mean	SD	Rank	
2.The nurse having to deal with distraught family members while still providing care for the patient.	2.91	1.21	1	3.09	1.31	1	8.99
21.The nurse having to deal with angry family members.	2.86	1.30	2	3.06	1.37	2	8.75
22.The family, for whatever reason, is not with the patient when he or she is dying.	2.82	1.29	3	3.02	1.29	4	8.52
5.Not enough time to provide quality end-of-life care because the nurse is consumed with activities that are trying to save the patient’s life.	2.72	1.43	4	2.94	1.41	5	8.00
1.Families not accepting what the physician is telling them about the patient’s poor prognosis.	2.59	1.10	8	3.03	1.29	3	7.85
19.Family members not understanding what “life-saving measures” really mean, i.e., that multiple needle sticks cause pain and bruising, that an ET tube won’t allow the patient to talk, or that ribs may be broken during chest compressions.	2.68	1.31	6	2.90	1.33	6	7.77
20.The nurse not knowing the patient’s wishes regarding continuing with treatments and tests because of the inability to communicate due to a depressed neurological status or due to pharmacologic sedation.	2.68	1.29	6	2.87	1.30	7	7.69
3.Intra-family fighting about whether to continue or stop life support.	2.54	1.12	10	2.87	1.25	8	7.29
6.Poor design of units which do not allow for privacy of dying patients or grieving family members.	2.55	1.44	9	2.70	1.47	9	6.89
10.No available support person for the family such as a social worker or religious leader.	2.54	1.64	10	2.70	1.66	9	6.86
14.Continuing treatments for a dying patient even though the treatments cause the patient pain or discomfort.	2.53	1.32	12	2.62	1.38	11	6.63
18.Being called away from the patient and family because of the need to help with a new admit or to help another nurse care for his/her patients	2.47	1.31	13	2.61	1.33	13	6.45
7.Unit visiting hours that are too restrictive.	2.47	1.41	13	2.59	1.43	15	6.40
15.Lack of nursing education and training regarding family grieving and quality end-of-life care.	2.40	1.36	15	2.62	1.37	11	6.29
4.The nurse knowing about the patient’s poor prognosis before the family is told the prognosis.	2.69	1.47	5	2.33	1.55	20	6.27
12.Continuing intensive care for a patient with a poor prognosis because of the real or imagined threat of future legal action by the patient’s family.	2.40	1.36	15	2.57	1.40	16	6.17
13.Pressure to limit family grieving after the patient’s death to accommodate a new admit to that room.	2.33	1.33	18	2.6	1.43	14	6.06
17.The unavailability of an ethics board or committee to review difficult patient cases.	2.36	1.45	17	2.52	1.46	17	5.95
9.Dealing with the cultural differences that families employ in grieving for their dying family member.	2.25	1.22	19	2.51	1.31	18	5.65
16.Physicians who won’t allow the patient to die from the disease process.	2.24	1.21	20	2.43	1.27	19	5.44
11.Employing life sustaining measures at the families’ request even though the patient had signed advanced directives requesting no such treatment.	2.11	1.31	21	2.32	1.37	21	4.90
26.When the nurses’ opinion about the direction patient care should go is not requested, not valued, or not considered.	1.97	1.21	22	2.19	1.31	22	4.31
24.Multiple physicians, involved with one patient, who differ in opinion about the direction care should go.	1.68	1.17	23	1.91	1.29	23	3.21
23.Physicians who are evasive and avoid having conversations with family members.	1.59	1.28	25	1.84	1.36	24	2.93
8.The patient having pain that is difficult to control or alleviate.	1.64	1.21	24	1.78	1.25	25	2.92
25.Continuing to provide advanced treatments to dying patients because of financial benefits to the hospital.	1.34	1.31	26	1.57	1.40	26	2.10

*SD* Standard Deviation; the perceived intensity score (PIS) mean for intensity multiplied by mean for frequency

### ICU nurses’ perceptions of supportive behaviors

Table 3 shows the perceived supportive behaviors of ICU nurses in end-of-life care. According to the results of PSBS, three of the top six behaviors related to team members: “having the physician meet in person with the family after the patient’s death to offer support and validate that all possible care was done (item 22)”; “having fellow nurses take care of your other patient(s) while you get away from the unit for a few moments after the death of your patient (item 15)”; “having the physicians involved in the patient’s care agree about the direction care should go (item 4)” The other three behaviors were associated with family members: “providing a peaceful, dignified bedside scene for family members once the patient has died (item 11)”; “having family members accept that the patient is dying (item 19)”; “allowing family members adequate time to be alone with the patient after he or she has died (item 12)” Behaviors with low perceived intensity scores included unrestricted visits, ethics committee members’ involvement and discussions with patients about dying. The intensity and frequency rankings of the top six supportive behaviors were basically the same.

**Table 3:** ICU nurses’ perceived supportive behaviors to end-of-life care

Supportive Behavior	Frequency score			Intensity score			PSBS
	Mean	SD	Rank	Mean	SD	Rank	
22.Having the physician meet in person with the family after the patient’s death to offer support and validate that all possible care was done.	3.19	1.38	1	3.35	1.34	2	10.69
11.Providing a peaceful, dignified bedside scene for family members once the patient has died.	3.06	1.44	2	3.36	1.44	1	10.29
15.Having fellow nurses take care of your other patient(s) while you get away from the unit for a few moments after the death of your patient.	2.99	1.43	3	3.16	1.41	5	9.45
19.Having family members accept that the patient is dying.	2.93	1.25	5	3.21	1.26	3	9.42
4.Having the physicians involved in the patient’s care agree about the direction care should go.	2.94	1.34	4	3.14	1.36	7	9.24
12.Allowing family members adequate time to be alone with the patient after he or she has died.	2.90	1.41	7	3.17	1.45	4	9.19
2.Having enough time to prepare the family for the expected death of the patient.	2.90	1.24	7	3.16	1.33	5	9.16
5.Having a unit schedule that allows for continuity of care for the dying patient by the same nurses.	2.91	1.47	6	3.10	1.46	9	9.03
13.Having a fellow nurse tell you that, “You did all you could for that patient,” or some other words of support.	2.87	1.32	9	3.11	1.35	8	8.93
9.Teaching families how to act around the dying patient such as saying to them, “She can still hear...it is OK to talk to her.”	2.79	1.42	10	3.04	1.39	10	8.48
17.Having family members thank you or in some other way show appreciation for your care of the patient who has died.	2.70	1.32	11	3.04	1.34	10	8.21
1.Having one family member be the designated contact person for all other family members regarding patient information.	2.63	1.37	12	2.98	1.46	12	7.82
20.After the patient’s death, having support staff compile all the necessary paper work for you which must be signed by the family before they leave the unit.	2.61	1.45	14	2.98	1.41	12	7.77
7.Having the family physically help care for the dying patient.	2.62	1.44	13	2.94	1.48	14	7.70
23.Having un-licensed personnel available to help care for dying patients.	2.52	1.46	16	2.91	1.47	15	7.32
16.Having a support person outside of the work setting who will listen to you after the death of your patient.	2.53	1.44	15	2.83	1.45	17	7.15
6.The nurse drawing on his/her own previous experience with the critical illness or death of a family member.	2.49	1.34	17	2.81	1.41	18	7.01
3.A unit designed so that the family has a place to go to grieve in private.	2.43	1.44	19	2.87	1.53	16	6.98
21.Physicians who put hope in real tangible terms by saying to the family that, for example, only 1 out of 100 patients in this patient’s condition will completely recover.	2.46	1.36	18	2.73	1.37	19	6.72
14.Having a fellow nurse put his or her arm around you, hug you, pat you on the back or give some other kind of brief physical support after the death of your patient.	2.43	1.37	20	2.73	1.40	19	6.65
10.Allowing families unlimited access to the dying patient even if it conflicts with nursing care at times.	2.33	1.27	21	2.60	1.37	22	6.06
18.Having an ethics committee member routinely attend unit rounds so they are involved from the beginning should an ethical situation with a patient arise later.	2.18	1.48	22	2.63	1.51	21	5.74
8.Talking with the patient about his or her feelings and thoughts about dying.	1.93	1.39	23	2.32	1.52	23	4.46

*SD* Standard Deviation; the perceived supportive behavior score (PSBS) mean for intensity multiplied by mean for frequency

### Responses to open-ended questions

Table 4 shows the nurses’ responses to open-ended questions. Seven participants answered the first open-ended question (Describe any missing obstacles in detail. Indicate how large each obstacle is and how frequently it occurs). Most of their answers were related to family, such as financial factors and interference therapy. Moreover, nurses believe there is little they can do. None of the participants answered the second open-ended question (Describe any missing supportive behaviors in detail. Indicate how large the support is and how frequently it occurs).

**Table 4:** Responses to open-ended questions

	Response to question one	Response to question three
family-related	Some family members with a medical background may interfere with the patient’s treatment. Family members deliberately make it difficult for the healthcare providers. Families whose hospitalization costs were covered more by medical insurance were more likely to prolong the lives of dying patients. The patient whose medical expenses are covered by government assistance programs is more likely to receive life-sustaining therapy	Prolong the time for family members to accompany the dying patients. I think families need to be given enough time to say goodbye to dying patients. Provide an area of privacy to help families vent their grieving emotions. Provide a separate space for family members to take care of the body of the deceased. Provide a space for family members to change the clothing worn by the deceased. It is not possible for family members to stay with the dying patient all the time, but we can allow family members to visit flexibly and increase the number of visits
healthcare providers- related	Because we had no systematic hospice training, we didn’t know what to do. There is little we can do at present. I didn’t know what to do or how to comfort the family members when they cried. Although we were with the dying patients, I did not know what they wanted to express, such as that they wanted to see thier family.	Healthcare providers need to listen to family members. Involve psychological consultants in the care of dying patients and their families. Healthcare providers should strengthen the knowledge of end-of-life care have enough ICU nurses.
others	End-of-life care is not popular enough. Chinese people avoid talking about the topic of “death”, we can not communicate with family members and terminal patients about it, which will cause unnecessary trouble.	Alleviate the pain of dying patients.

Question one: Describe any missing obstacles in detail. Indicate how large each obstacle is and how frequently it occurs

Question three: If you had the ability to change just one aspect of the end-of-life care given to dying ICU patients, what would it be?

Thirty-one participants answered the third open-ended question (If you had the ability to change just one aspect of the end-of-life care given to dying ICU patients, what would it be?). Their responses mainly focused on three aspects: companionship, space and psychological support. In addition, nurses want to be trained in end-of-life care.

## Discussion

This study quantified the intensity and frequency of the barriers to and supportive behaviors of end-of-life care of ICU nurses in mainland China. The results showed that lack the knowledge of end-of-life care, can’t accompany patients, take inappropriate actions, and their financial situation were the prominent factors, and these significant factors were all related to family. Moreover, family-related supportive behaviors were prominent in end-of-life care, which included: accompany the dying patients, accept the patients is dying and have a dignity environment. Additionally, it remarkable that nurses almost all concern care after death.

Family-related factors are important considerations in end-of-life care by ICU nurses in China. However, in other studies, key factors focused on patients’ comfort and dignity and healthcare providers’ lack of education [28–31]. Under the Confucian culture of “family-oriented”, the family plays an essential role in medical treatment [32]. Family members are the primary caregivers and are closest to the patient. The results of this study suggest that family companionship is a major factor affecting end-of-life care in the ICU. The presence of family gives the dying patient a sense of satisfaction and security [12], the company of family members will make patients feel close without strangeness. In mainland China, family relationships are emphasized and take priority over the patient’s rights [20]. Medical decisions are often made by family members, even when the patient is alert and oriented [33]. However, a family’s lack of knowledge affects their decision-making. They often do not understand the meaning of life-sustaining therapy and continue to advocate for the use of invasive treatment. A study showed the family often did not correctly understand the risk of treatments, so they would continue to choose painful treatments rather than end-of-life care [34]. Although making medical decisions for patients is a complex process, structured communication tools can be used to clarify the patient’s wishes and set care goals [35]. A study showed the family often did not correctly understand the risk of treatments, so they would continue to choose painful treatments rather than end-of-life care [34]. Healthcare providers should avoid using complex medical terms to communicate with family members. In mainland China, living wills has been gradually promoted, which has received enough attention, but no relevant laws have been established. After all, there is still a long process from attracting attention to establishing relevant laws [32].

In addition, nurses considered that one of the factors influencing end-of-life care was the family’s financial situation, which would influence family decision-making. Some poor families often abandon the treatment of a family member due to medical costs, while those with adequate finances often persisted in using aggressive treatment for dying patients. A qualitative study in China also found similar results [36]. In mainland China, the patient whose medical expenses are covered by government assistance programs is more likely to receive life-sustaining therapy. Beckstrand’s study also found that the family might use legal means to keep the patient alive in order to receive the patient’s welfare checks [16].

Another family-related barrier is that family members who take inappropriate actions can put pressure on the nurse. In this study, nurses described that family members with a medical background may sometimes interrupt the patient’s treatments. If the healthcare provider did not follow the directives of the family, this could cause medical disputes. A strained nurse-patient relationship places pressure on nurses. The nurse may feel stressed when dealing with the family member’s emotional reactions. Kisorio’s study showed that family members who responded with anger and madness were more likely to be trouble makers [31].

In this study, it is remarkable that nurses’ supportive behaviors almost all concern care after death. Although death is considered a bad thing in Chinese culture, care after death is regarded as very important [25]. On the one hand, nurses want to reduce the family’s regret, on the other hand, they want the deceased to achieve a good death. Care after death is also part of a good death. The barriers found in our study almost all concern behaviours before death. This suggests that the supportive behaviours found are helping to cope with the trauma of the family after death, whereas the barriers keep nurses from providing good end-of-life care for the patient. As the nurse responded the most wanted to change, they want to provide a private environment for the family to provide personal attention to their loved one’s body. In traditional Chinese culture, the body of a deceased patient should be clean, and the family members dress the deceased in funeral clothes, so that family members think the deceased died peacefully.

Nurses reflected on their role and felt there was not much they could do for dying patients because they lacked time and did not know how to help them. In mainland China, the number of ICU nurses is insufficient, and their workload is heavy. The same finding was reported in Australia [37] and Hong Kong [20]. They lack time to provide comprehensive care for their patients, especially those who are dying. And they don’t know exactly what end-of-life care is, so they can be confused. Although nurses have been caring for dying patients for a long time, they do not think they understand the wishes of dying patients. This is contrary to other studies [28–30]. This may be because many patients are in a coma or delirium. On one hand, nurses should communicate with families frequently to clarify the wishes of dying patients. On the other hand, nurses should be trained in end-of-life care.

Teamwork is another important aspect in the development of end-of-life care in ICU settings. A previous study came to a similar conclusion [37]. Results indicated that having a physician involved in directing care was a major supportive behavior. Studies in Asia showed that physicians unwilling to discuss end-of-life care issues [38]. A physician plays a critical role in implementing end-of-life care, for example, leading family meetings, maintaining patients’ right to know and choice, and coordinating patients’ family relations [11]. Communication and cooperation among healthcare team members can help reduce differences, such as those involving treatments options. Additionally, efficient teamwork can reduce medical disputes. In Chinese healthcare settings, patients and families seem to trust physicians more than nurses. Having the physicians answer the family’s questions, provide compassionate and informative communication, and provide emotional and psychological support for family members may make it easier for them to accept the patient’s poor prognosis [39]. In addition, training of end-of-life care communication should be provided to ICU nurses since nurses are the communication pivot in ICUs.

## Strength and limitations

There are several strengths to the current study, including a 53% response rate, which improves the content validity and clinical sensitivity of the questionnaire. Participants worked in ICUs in large cities in various regions of China which allows for the generalizability of the findings to diverse settings. The study identified the prominent barriers and supportive behaviors, which provides a basis for developing corresponding solutions. This will drive the development of end-of-life care and possibly improve its quality. A limitation of this study is that it is a cross-sectional study which focused on only ICU nurses’ perceptions of end-of-life care, and did not involve other members of the healthcare team. The perspectives of these team members are also critical in identifying other barriers and supportive behaviors as well as cultural issues in mainland China. In addition, this study was conducted in the early stages of COVID-19, and in special circumstances, nurses may be in a state of psychological stress, and the results may be different from normal.

## Conclusion

Results of this study suggest that family-related factors and teamwork issues are prominent in the development of end-of-life care in ICU settings. In mainland China, it is necessary to popularize end-of-life care education to the public and train end-of-life care professionals. There is also a need to explore family members’ perceptions of barriers to and supportive behaviors of end-of-life care.

## Supplementary Information

**Additional file 1.**

## Data Availability

All data generated or analysed during this study are included in this published article [and its supplementary information files].

## Abbreviations

ICU: Intensive care unit
EOLC: End-of-life care
PIS: The perceived intensity score
PSBS: The perceived supportive behavior score
